# Coexisting Germline *CHEK2* and Somatic *BRAF*^V600E^ Mutations in Papillary Thyroid Cancer and Their Association with Clinicopathological Features and Disease Course

**DOI:** 10.3390/cancers11111744

**Published:** 2019-11-07

**Authors:** Danuta Gąsior-Perczak, Artur Kowalik, Agnieszka Walczyk, Monika Siołek, Krzysztof Gruszczyński, Iwona Pałyga, Estera Mikina, Tomasz Trybek, Janusz Kopczyński, Ryszard Mężyk, Stanisław Góźdź, Aldona Kowalska

**Affiliations:** 1 Endocrinology, Holycross Cancer Centre, S. Artwińskiego St. 3, 25-734 Kielce, Poland; a.walczyk@post.pl (A.W.); iwonapa@tlen.pl (I.P.); esterami@tlen.pl (E.M.); trytom1@tlen.pl (T.T.); aldonako@onkol.kielce.pl (A.K.); 2 Molecular Diagnostics, Holycross Cancer Centre, S. Artwińskiego Str. 3, 25-734 Kielce, Polandgruszczynski.k@wp.pl (K.G.); 3 Genetic Clinic, Holycross Cancer Centre, 25-734 Kielce, Poland; monika.siolek@wp.pl; 4 Surgical Pathology, Holycross Cancer Centre, S. Artwińskiego Str. 3, 25-734 Kielce, Poland; janusz_kopczynski@yahoo.com; 5 Cancer Epidemiology, Holycross Cancer Center, 25-734 Kielce, Poland; Ryszard.Mezyk@gmail.com; 6 The Faculty of Health Sciences, Jan Kochanowski University, IX Wieków Kielc Av. 19, 25-319 Kielce, Poland; stanislawgozdz1@gmail.com; 7 Clinical Oncology, Holycross Cancer Centre, S. Artwińskiego Str. 3, 25-734 Kielce, Poland

**Keywords:** papillary thyroid cancer, *BRAF* mutation, *CHEK2* mutation, risk stratification, response to therapy

## Abstract

*BRAF*^V600E^ is the most common somatic mutation in papillary thyroid carcinoma (PTC) and the majority of evidence indicates that it is associated with an aggressive clinical course. Germline mutations of the *CHEK2* gene impair the DNA damage repair process and increase the risk of PTC. Coexistence of both mutations is expected to be associated with poorer clinical course. We evaluated the prevalence of concomitant *CHEK2* and *BRAF*^V600E^ mutations and their associations with clinicopathological features, treatment response, and disease course in PTC patients. The study included 427 unselected PTC patients (377 women and 50 men) from one center. Relationships among clinicopathological features, mutation status, treatment response, and disease outcomes were assessed. Mean follow-up was 10 years. *CHEK2* mutations were detected in 15.2% and *BRAF*^V600E^ mutations in 64.2% patients. Neither mutation was present in 31.4% cases and both *BRAF*^V600E^ and *CHEK2* mutations coexisted in 10.8% patients. No significant differences in clinicopathological features, initial risk, treatment response, or disease outcome were detected among these patient groups. *CHEK2* mutations were significantly associated with older age, while *BRAF*^V600E^ was significantly associated with older age and extrathyroidal extension. The coexistence of both mutations was not associated with more aggressive clinicopathological features of PTC, poorer treatment response, or disease outcome.

## 1. Introduction

Papillary thyroid carcinoma (PTC) is the most common histopathologic type of thyroid cancer, accounting for approximately 80%–85% of all thyroid cancers, and its incidence is rapidly increasing across the world [1,2,3]. The biological behavior of PTC varies widely, from slow growing microcarcinomas to invasive cancers that can lead to death. 

Constitutive activation of the mitogen activated protein kinase (MAPK) pathway is key to the oncogenic processes underlying PTC and can be initiated by various genetic events. In approximately 70% of cases, the somatic activating point mutation, *BRAF*^V600E^, RET/PTC rearrangements, and *RAS* mutations are responsible for abnormal activation of the MAPK pathway [4,5]. The *BRAF*^V600E^ mutation has been reported to occur in 27%–87% of PTC cases [6,7,8] and is considered to contribute to oncogenic transformation in thyroid cancer, potentially functioning to silence specific tumor suppressor and differentiation genes by methylation, thereby causing PTC cells to be insensitive to radioiodine treatment, which can lead to persistent or recurrent disease [9]. 

Checkpoint kinase 2 (*CHEK2*) is a tumor suppressor gene that encodes the serine/threonine protein kinase CHK2, a key regulator of the cell cycle that also influences apoptosis and cell aging [10,11]. CHK2 is activated by the ataxia telangiectasia mutated (ATM) serine-threonine kinase when double-stranded DNA breaks are detected. ATM also activates several other proteins, including P53, to induce cell cycle arrest in the G1 and G2 phase, or stimulate DNA repair or apoptosis [10,12]. Mutations in genes encoding these DNA damage response proteins (ATM, CHK2, and p53) lead to neoplastic transformation of cells. *CHEK2* mutations, which occur in various sporadic cancers, predispose individuals to several types of hereditary malignancy, including thyroid cancer [13,14,15]. According to The Cancer Genome Atlas [16], mutations in *CHEK2* are present in only 1.2% of patients with PTC, and are not mutually exclusive with other mutations involved in the MAPK signaling pathway, although frequencies of *CHEK2* mutations ranging from 0% to 15.6% have been reported in patients with PTC [13,16,17,18,19]. Moreover, defects in DNA repair may be one mechanism underlying the features of more aggressive PTC [16]. 

Four founder *CHEK2* germline mutations have been detected in Poland: Three protein truncating variants (1100delC, IVS2+1G > A, and del5395) and a fourth, missense variant (I157T), which causes an isoleucine to threonine amino acid change [20]. All four of these alleles are associated with an increased risk of various cancers, including PTC [13,21]. Of these, truncating mutations of *CHEK2* are associated with a greater risk (2–3 times) of breast, prostate, and stomach cancers, as is the missense mutation, I157T (1.5 times), whereas in kidney and colorectal cancer, only the missense variant, but not the truncating variant, appears to be pathogenic [13,17,22]. Hereditary mutations in *CHEK2* increase the risk of PTC in carriers. In our previous study, we showed that 73/486 (15.6%) patients with PTC and 28/460 (6.0%) healthy controls had one of four mutations in *CHEK2*. Further, truncating *CHEK2* mutations (1100delC, IVS2+1G > A, and del5395) were associated with higher risk of thyroid cancer (odds ratio [OR] = 5.7; *p* = 0.006) than were missense mutations (c.470T > A, I157T, and rs17879961) (OR = 2.8; *p* = 0.0001) [17]. 

There is a discussion in the medical literature regarding the influence of the *BRAF*^V600E^ mutation on the clinical and pathological features of PTC, with conflicting data reported [7,9,23,24,25,26,27]. In this study, we aimed to assess whether the coexistence of both somatic *BRAF*^V600E^ and germline *CHEK2* mutations in PTC is associated with a poorer disease course. We analyzed samples from 427 patients with PTC treated in single center in Poland for these mutations, assessed the prevalence of their coexistence, and determined whether PTC in individuals with these two types of mutation is associated with specific clinicopathological features, primary treatment responses, or disease outcomes.

## 2. Materials and Methods

### 2.1. Patients and Study Design

The study group consisted of patients from a single center who had undergone total thyroidectomy or lobectomy, treated between 2000 and 2015, who were included in the study during follow-up visits at the Endocrinology Outpatient Department between 2011 and 2015. The initial group comprised 468 unselected patients with PTC from whom blood samples were taken for *CHEK2* mutation screening. Archived paraffin-embedded blocks of primary tumor tissue were obtained from 455 of the 468 patients for evaluation for the presence of the *BRAF*^V600E^ mutation. Twenty-eight patients were excluded from the study due to degraded DNA. Finally, 427 patients were included in the study. Clinicopathological data were available from all analyzed cases and were subjected to retrospective analysis. Postoperative Tumor-Node-Metastasis (TNM) staging of all included patients was reclassified according to the most recent (8th) edition of the American Joint Committee on Cancer (AJCC)/Union for International Cancer Control (UICC) TNM staging system, and the American Thyroid Association (ATA) modified initial risk stratification system (low, intermediate, and high risk of recurrence) was used [28,29].

Clinicopathological characteristics at diagnosis, including TNM stages pNx and Mx, were analyzed in detail, with pNx clinically reclassified as N0b or N1, while Mx was reclassified as M0 or M1, according to the 8th edition of the AJCC/UICC TNM staging system. Suspicious pNx changes found on postoperative ultrasound were verified by fine-needle biopsy, with evaluation of thyroglobulin from the aspirate, as previously described [7]. Patients were divided into four groups, according to the type of mutation: *BRAF*^V600E^ only, *CHEK2* only, both *BRAF*^V600E^ and *CHEK2* mutations, and neither *BRAF*^V600E^ nor *CHEK2* mutations—*BRAF*^V600E^ wild type and *CHEK2* wild type (*BRAF*^V600E^/*CHEK2* WT). The following clinicopathological features were analyzed, and their relationship with the evaluated mutations was determined: sex, age at diagnosis, tumor diameter, PTC histologic variant, multifocality, lymph node metastases, distant metastases, extrathyroidal extension, vascular invasion, initial risk stratification, response to initial treatment, and disease outcome (remission, persistent disease, and death). The follow up results were finally concluded on 31 October 2018.

The study was approved by the Bioethics Committee at the Świętokrzyska Chamber of Physicians on 26 March 2013 and 28 June 2018 (ethic code: 2/2013 and 58/2018), and patients provided written informed consent to participate in the study. 

### 2.2. Management and Follow-Up Protocols

All patients enrolled in the study underwent primary surgical treatment. The scope of treatment included lobectomy, total thyroidectomy, or complete thyroidectomy with central compartment lymphadenectomy. The surgical treatment procedures conducted in our center have been described previously [30]. All patients with disease stage more advanced than pT1aN0-xM0 were eligible for radioactive iodine (I-131) treatment. Postoperative assessment reports, including laboratory and imaging analyses, whether patients were treated with I-131 or not, all examinations and all procedures evaluating response to initial therapy, were recorded according to ATA classifications (excellent, indeterminate, and biochemically and structurally incomplete), and reclassified according to the ATA delayed stratification system, which was accepted in our center, as previously described in detail [31,32]. Risk stratification was repeated at multiple treatment stages, and response categories were updated according to the ATA guidelines during follow-up [29]. 

### 2.3. End of Follow-Up with Oncological Assessment on 31 October 2018

Information about the health of patients was obtained from medical records, and patients were assigned (according to the latest ATA guidelines) [29] into the following groups: No evidence of disease (NED), persistent disease (biochemically and structurally), recurrent disease, and death. No evidence of disease was defined as suppressed thyroglobulin (Tg) < 0.2 ng/mL, stimulated Tg < 1 ng/mL and no structural disease in the absence of interfering anti-Tg antibodies (TgAb). Biochemically persistent disease was defined as suppressed Tg ≥ 0.2 ng/mL or stimulated Tg ≥ 1ng/mL, persisting/increasing TgAb levels in the absence of structural disease within 12 months after initial therapy (surgery + I131). Structurally persistent disease was defined as presence of neoplastic changes on ultrasound or on whole body scan with any Tg level within 12 months after initial therapy (surgery + I131). Recurrent disease was defined as biochemical or structural evidence of disease after a period of NED for at least 12 months after initial therapy (surgery + I131). In patients not treated with I131, biochemical assays such as serum Tg and TgAb levels were not used as criteria for recurrent/persistent disease [33]. 

### 2.4. Detection of BRAF^V600E^ Mutation

DNA was extracted from archived tumor specimens in paraffin-embedded blocks, and the presence of the *BRAF*^V600E^ mutation was evaluated by real-time PCR (qPCR). The methods used for DNA isolation and genotyping, and the diagnostic algorithm applied for molecular research in our center, have been described in detail in our previous publications [34,35].

### 2.5. Detection of CHEK2 Mutations

#### 2.5.1. DNA Isolation

DNA was isolated from whole blood samples (100 μl), collected in EDTA tubes, within 12 h of collection, using the Micro AX Blood Gravity Kit (A&A Biotechnology, Gdańsk, Poland). DNA samples were eluted in 120 μl of buffer E.

#### 2.5.2. CHEK2 Genotyping

The p.Ile157Thr (I157T) mutation in exon 3 was genotyped using the TaqMan PCR method with the following primers and probes:

CHEK2_EK3_F: GCTGGTAATTTGGTCATTGT
CHEK2_EK3_R: CCTACAAGCTCTGTATTTCAAA
I157T_T: 6-FAM CTTCTATGTATGCAATGTAAGAGTT–TAMRA
I157T_C: VIC CTTCTATGTATGCAGTGTAAGAGTT–TAMRA

The IVS2+1G > A mutation in intron 2 was genotyped using allele-specific PCR with the following primers:

F1MUT: CAAGAAACACTTTCGGATTTTCATGA
F2 CONTROL: ACAAAAGCTGTGAATATTGCTTTGATGA
R1 WT: TCCTAGATACATGGGTATTCATTACCGAC
R2 CONTROL: GTGGGAAAATATCTAAAAACAATGACCAA

yielding the following products: control, 161 bp (F2 CONTROL & R2 CONTROL); wild type (WT), 121 bp; IVS2+1G > A mutation, 95 bp. 

The delC1100 mutation in exon 10 was genotyped by allele-specific PCR using the following primers:

CHEK2_1100delC_3_F: GAACTTCAGGCGCCAAGT
CHEK2_1100delC_3_R: TAGAAACTGATCTAGCCTACGTGT
CHEK2_1100delC_3_Mut: CAAAATCTTGGAGTGCCCAAAATAAT

to yield the following products: control, 194 bp; delC1100 mutation, 128 bp.

An extensive deletion (del5395) involving exons 10 and 11 was detected by allele-specific PCR using the following primers [36]:

CHEK_ek10_F_1: TGTAATGAGCTGAGATTGTGC
CHEK_ek10_F_2: GTCTCAAACTTGGCTGCG
CHEK_ek10_R_1: CAGAAATGAGACAGGAAGTT
CHEK_ek10_R_2: CTCTGTTGTGTACAAGTGAC

which generated 522 and 397 bp WT products in the absence of the mutation. Where the del5395 mutation was present, an additional product of 443 bp was amplified by the CHEK_ek10_F_1 and CHEK_ek11_R_1 primers.

#### 2.5.3. Sanger Sequencing

All mutations (I157T, IVS2+1G > A, and delC1100) detected by TaqMan PCR and allele-specific PCR were confirmed by capillary sequencing. PCR amplification products, purified by incubation with 10 U of exonuclease I (EN 0582) and 1 U of phosphatase Fast-AP (EF 0651) (both from ThermoFisher Scientific, Waltham, MA) for 15 min at 37 °C, followed by 20 min at 80 °C, were used as template for Sanger sequencing reactions. Sequencing reactions were performed using forward and reverse sequence-specific primers (CHEK2_EK3_F & CHEK2_EK3_R; F2 CONTROL & R2 CONTROL; CHEK2_1100delC_3_F & CHEK2_1100delC_3_R) and an ABI PRISM Big Dye Terminator Kit, version 3.1 (catalogue number: 4337450, Applied Biosystems/ThermoFisher Scientific), according to the manufacturer’s instructions. Sequencing results were analyzed using a 3130 Capillary Sequencer (Applied Biosystems/ThermoFisher Scientific). The sequences generated were compared with the reference sequence (NM_007194.4) using the NCBI BLAST Nucleotide tool. We decided to use Sanger sequencing instead of next-generation sequencing tests (NGS) in the analysis because the sensitivity of Sanger sequencing is at the level of 20% of allelic frequency so it is sufficient to detect germline mutation which are at the level of about 50% allelic frequency [37].

### 2.6. Statistical Analyses

Continuous variables are presented as descriptive statistics (min, max, average, standard deviation, median, and quartiles). Comparisons between two groups with normally and non-normally distributed data were evaluated using the t-test and the Mann–Whitney test, respectively. Categorical variables are presented as frequencies and percentages. The relationship between two-way classification factors were made using the chi-square test. Statistical significance was assumed when p-values were less than 0.05. The Bonferroni correction was applied for multiple comparisons and the *p*-value < 0.0125 [0.05/4] is considered as significant. Analyses were performed using MedCalc Statistical Software version 18.5 (MedCalc Software bvba, Ostend, Belgium; http://www.medcalc.org; 2018).

## 3. Results

### 3.1. Baseline Characteristics

The clinicopathological features of patients with PTC, molecular status of the *BRAF*^V600E^ and *CHEK2* mutations, categories of response to initial treatment, and disease outcomes for all 427 cases are summarized in Table 1.

In the study group of 427 unselected Caucasian patients (median age, 50; range, 15–76 years), the majority (377, 88.3%) were women and under 55 years of age (278, 65.1%). The mean and standard deviation tumor diameter for all patients was 13.5 ± 12.6 mm (range, 1.0–80 mm). Primary tumor stage was pT1 in 336 patients (78.7%), and the dominant classic histological variant of PTC was detected in 353 patients (82.7%). Microscopic extrathyroidal extension was found in 125 (29.3%) whilst none of the patients had gross extrathyroidal extension. Vascular invasion was found in 18 (4.2%), histologically verified metastases to lymph nodes in 48 (11.2%), and distant metastases in 4 (0.9%) patients. Four patients (0.9%) had more advanced stage (stage III/IV) tumors, according to the updated 8th AJCC/TNM staging system, and multifocality was detected in 100 patients (23.4%). 

The *BRAF*^V600E^ mutation was found in 274 (64.2%) patients, and in 228 (53.4%) only this mutation was detected. *CHEK2* gene mutations were found in 65 (15.2%) patients and in 19 (4.4%) patients who did not have the *BRAF*^V600E^ mutation. The dominant mutation in the *CHEK2* gene was the I157T missense alteration, which was found in 56 (86.2%) mutation-positive patients. The *BRAF*^V600E^ and *CHEK2* mutations coexisted in 46 (10.8%) patients, while neither *BRAF*^V600E^ nor *CHEK2* mutations were found in 134 (31.4%) patients. 

One hundred and fifty-one patients (35.4%) were classified as intermediate risk and 11 (2.6%) as high risk, according to the latest ATA guidelines [29]. I-131 treatment was administered to 326 (76.3%) patients, at doses from 1100 to 3700 MBq, depending on TNM stage. The remaining 101 patients (23.7%) had disease restricted to unifocal PTC in the thyroid gland, with diameter ≤ 1 cm, without nodal or distant metastases (pT1aN0-xM0), and did not receive I-131 treatment. In the entire study group, 363 (85%) patients exhibited an excellent response to primary treatment. Recurrence after a period of NED was observed in four patients (0.9%). The median follow-up for the study group was 10 years (range, 3–18 years). There were no deaths from cancer or other causes. At the end of follow-up, 19 patients (4.4%) presented with features of a biochemically and structurally persistent disease.

### 3.2. Clinical Characteristics of Patients with PTC Carrying Two CHEK2 Mutations

Of the 427 patients, three (0.7%) carried two *CHEK2* mutations; one woman was a carrier of both IVS211G > A and I157T, and one woman and one man had homozygous I157T mutations (Table 2). All three (100%) of these patients with biallelic *CHEK2* mutations also carried the *BRAF*^V600E^ alteration. Clinicopathological features, response to treatment, and disease outcome in these three patients were similar to those of patients with single *CHEK2* mutations. The average age of patients diagnosed with biallelic *CHEK2* mutations was 61 years (range, 57–66 years). All patients had the classic histological variant of PTC. The stage of the primary focus was described as T1 in two of the three patients and T2 in one patient, who was a homozygous I157T mutation carrier. Multifocality was detected in two patients, who were both homozygous for the I157T mutation. Lymph node metastases were identified in one patient who had the homozygous I157T mutation. None of the patients had extrathyroidal extension or vascular invasion. Two of the three patients had TNM stage I and one had TNM stage II tumors. One patient homozygous for the I157T mutation was classified into the intermediate risk group, and the remaining two patients were classified into the low risk group, according to the latest ATA guidelines. Treatment with I-131 (2700 MBq) was administered to all patients, and excellent responses to initial treatment were obtained in all three patients. None of the patients had recurrence after a period of NED, had distant metastases, or died from cancer or other causes.

### 3.3. Associations of BRAF^V600E^ Mutation Alone, CHEK2 Mutation Alone, and Coexisting BRAF^V600E^ and CHEK2 Mutations on Clinicopathological Characteristics, Relative to BRAF^V600E^/CHEK2 WT

The results of screening for *BRAF*^V600E^ and *CHEK2* mutations in 427 patients with PTC are presented in Table 3, along with the relationships between individual clinicopathological features and mutation groups (*BRAF*^V600E^ alone, *CHEK2* alone, coexisting *BRAF*^V600E^ and *CHEK2*, and *BRAF*^V600E^/*CHEK2* WT). There were no significant associations between any groups and the following characteristics: Sex, tumor size, multifocality, lymph node metastases, vascular invasion, and more advanced clinical stage of cancer. Patients with only *BRAF*^V600E^ or only *CHEK2* mutations were significantly older at diagnosis than those without any mutations (*p* = 0.016 and *p* = 0.028, respectively); however, analysis to determine whether the categories “<55 years of age” and “≥55 years of age” were prognostic factors, according to TNM, demonstrated no significant difference between the groups: “*BRAF*^V600E^/*CHEK2* WT vs. *BRAF”* (*p* = 0.099), “*BRAF*^V600E^/*CHEK2* WT vs. *CHEK2” (p* = 0.094), and “*BRAF*^V600E^/*CHEK2* WT vs. *BRAF*^V600E^ + *CHEK2” (p* = 0.175). Extrathyroidal extension was significantly more frequent in patients with only the *BRAF*^V600E^ mutation than in those *BRAF*^V600E^/*CHEK2* WT (*p* = 0.039). Distant metastases were significantly more common in the group of patients *BRAF*^V600E^/*CHEK2* WT than in those with the *BRAF*^V600E^ mutation alone (*p* = 0.009). There were also statistical differences in the histological variants of PTC among the groups (*BRAF*^V600E^/*CHEK2* WT vs. *BRAF, p* = 0.003; *BRAF*^V600E^/*CHEK2* WT vs. *CHEK2, p* = 0.027; *BRAF*^V600E^/*CHEK2* WT vs. *BRAF*^V600E^ + *CHEK2, p* = 0.101). The classic histological variant of PTC was observed more frequently in patients with *BRAF*^V600E^ (86.4%), *CHEK2* (84.2%), and both mutations together (89.1%), than in those *BRAF*^V600E^/*CHEK2* WT (73.9%). Follicular PTC was more common in patients without *BRAF*^V600E^/*CHEK2* WT (20.9%) relative to those carrying the *BRAF*^V600E^ (11.8%) and *CHEK2* (10.5%) mutations, or both (8.7%). Moreover, other aggressive PTC variants were more frequently observed in the group with *BRAF*^V600E^/*CHEK2* WT (5.2%) than in the *BRAF*^V600E^ alone group (0.9%). By contrast, no aggressive PTC variants were observed in patients with *CHEK2* mutations alone or those with both mutations.

### 3.4. Associations of BRAF^V600E^ Mutation Alone, CHEK2 Mutation Alone, Coexisting BRAF^V600E^ and CHEK2 Mutations, and BRAF^V600E^/CHEK2 WT with Response to Therapy and Final Disease Outcome

Risk of recurrence, according to the ATA initial risk stratification system, treatment response, and disease outcome were assessed according to type of mutation (*BRAF*^V600E^ alone, *CHEK2* alone, coexisting *BRAF*^V600E^ and *CHEK2*, and BRAF^V600E^/*CHEK2* WT) (Table 3). There were no significant relationships between the different groups, in terms of intermediate and high risk of recurrence, I-131 treatment, poorer response to initial treatment (indeterminate, biochemically and structurally incomplete), and disease outcome (persistent disease, death). Recurrence after a period of NED was observed in 1.8% (4 of 228) patients carrying the *BRAF*^V600E^ mutation alone and in no BRAF^V600E^/*CHEK2* WT patients (*p* = 0.124).

### 3.5. Associations of Coexisting BRAF^V600E^ and CHEK2 Mutations Compared with BRAF^V600E^ Mutation Alone with Clinicopathological Characteristics, Response to Therapy, and Disease Outcome 

Clinicopathological features, risk of recurrence (according to the ATA initial risk stratification system), response to therapy, and disease outcome were assessed in patients with coexisting *BRAF*^V600E^ and *CHEK2* mutations compared with the *BRAF*^V600E^ mutation alone (Table 3). There were no significant differences between *BRAF*^V600E^ + *CHEK2* and *BRAF*^V600E^ mutation alone in sex, age at diagnosis, tumor size, PTC histological variant, multifocality, lymph node metastases, distant metastases, extrathyroidal extension, vascular invasion, or more advanced stage of cancer. There were also no significant relationships between these groups in terms of intermediate and high risk of recurrence, I-131 treatment, poorer response to initial treatment (indeterminate, biochemically and structurally incomplete), and disease outcome (persistent disease, death). Recurrence after a period of NED was observed in 1.8% (4 of 228) of patients with the *BRAF*^V600E^ mutation alone and in no patients with coexisting *BRAF*^V600E^ and *CHEK2* mutation (*p* = 0.366).

## 4. Discussion

Excessive activation of the MAPK pathway has a major role in PTC oncogenesis, and the activating *BRAF*^V600E^ point mutation is considered the most important contributor to this process [38]. In the present study, the overall frequency of the *BRAF*^V600E^ mutation was 64.2%, consistent with previous reports of mutation rates in the range 27%–87% [6,7,8,16]. The large range of reported mutation frequencies may reflect the histological heterogeneity of PTC, the heterogeneity of tumor mutation status, the geographical area where research was conducted, epidemiological factors, and particularly patient age and the use of different genetic research methods, with varying levels of sensitivity [9,34,39,40]. The correlation between the *BRAF*^V600E^ mutation and more aggressive clinicopathological features, recurrent disease, persistent disease, and risk of death remains controversial. Numerous studies have reported positive correlations between the *BRAF*^V600E^ mutation and these features [9,23,24,25,27,41,42]; however, other investigations have detected no such relationship [43,44,45,46,47]. Our data indicate that *BRAF*^V600E^ was associated with a higher risk of extrathyroidal extension (*p* = 0.039). Further, patients with the *BRAF*^V600E^ mutation were significantly older than those without mutations (*p* = 0.016), similar to previous reports [7,27,48,49]; nevertheless, analysis of whether the variables “<55 years of age” and “≥55 years of age” were prognostic factors, according to the 8th edition of the AJCC/UICC TNM system for differentiated thyroid carcinoma (DTC) [28], revealed no significant difference between the groups (*p* = 0.099). In addition, we did not detect a relationship between *BRAF*^V600E^ and the presence of cervical lymph node metastases at diagnosis, consistent with our previous study [7] and other studies [26,27,47,50,51]; conversely, there are reports in the literature where an association between these variables has been demonstrated [9,23,52]. 

No relationship between *BRAF*^V600E^ and distant metastases at diagnosis (the strongest prognostic factor for unfavorable outcome in DTC) was demonstrated in any of the PTC patients in this study, similar to previous reports [27,45,47]. M1 status is also included as an independent predictor of high risk of recurrence in the recently modified ATA stratification system [29]. According to previous studies [7,42,53], *BRAF*^V600E^ is more common in the classic PTC histological subtype than in follicular thyroid cancer or other types of PTC. Follicular thyroid carcinoma and aggressive variants of PTC are more frequent in patients with absence of *BRAF*^V600E^ [7,53]. There was no significant association between intermediate or high risk of recurrence and *BRAF*^V600E^ status, nor was this mutation correlated with treatment response or final disease outcome in this study. Recurrence after a period of NED occurred in 1.8% of *BRAF*^V600E^-positive patients with risk of recurrence initially determined as intermediate (according to the ATA system), while persistent disease (biochemically, structurally) was not significantly more common in patients with *BRAF*^V600E^. Our results are consistent with the findings of Kowalska et al., Daliri et al., and Nair et al. [7,54,55]; however, despite growing evidence regarding the lack of a direct effect of *BRAF*^V600E^ on risk of relapse, the latest ATA guidelines [29] recommend taking *BRAF*^V600E^ and/or *TERT* promoter mutation status into account during continuous re-evaluation of recurrence risk. *BRAF*^V600E^ mutation should be analyzed together with other molecular and clinicopathological prognostic factors for improved risk stratification [29].

Our results indicate that occurrence of the *BRAF*^V600E^ somatic mutation does not correlate with poor prognosis, apart from some adverse clinicopathological features. Therefore, we analyzed the coexistence of *BRAF*^V600E^ somatic mutation with less well-studied germline mutations in the *CHEK2* suppressor gene. In this study, the incidence of *CHEK2* mutations was 65/427 (15.2%), which is comparable to our previous findings [17]. By contrast, other studies by Fayaz et al. and Alzahrani et al. in Middle Eastern populations identified no *CHEK2* mutations, perhaps due to ethnic or geographical differences [18,19]. In a study reported by Wójcicka et al. in 2014, the variant I157T was identified as a risk factor for PTC (OR = 2.2, *P* = 2.37e-10) [56], using data from a large group of patients with PTC (n = 1781) and healthy control subjects (n = 2081). A study by Kaczmarek-Ryś et al. in 2015, including 602 patients with DTC and 829 healthy control subjects, showed that heterozygosity for the I157T variant is associated with an almost 2-fold increase in the risk of developing DTC (OR = 1.81, *p* = 0.004), while the homozygosity increased the risk of PTC almost 13-fold in women (OR = 12.81, *p* = 0.019); however, the latter relationship was not observed in men [15]. Several studies have demonstrated associations of mutations in *CHEK2* with clinicopathological features of PTC. In the present study, patients with *CHEK2* mutations were significantly older (mean age, 52.8 years) than those without any mutations (*p* = 0.028); however, analysis of data stratified by the variables “<55 years of age” and “≥55 years of age” to determine whether they are prognostic factors, similar to the *BRAF*^V600E^ mutation, indicated no significant difference (*p* = 0.094). Similar to the findings of Kaczmarek-Ryś et al. [15], we identified an association between the I157T allele and age at diagnosis, with the I157T allele more frequent in the patients with DTC between 51 and 60 years of age (*p* = 0.016). In the present study, *CHEK2* mutations were more common in patients with classic PTC than in those with variant PTC subtypes. We did not detect any significant relationships between *CHEK2* mutations and other aggressive clinicopathological features, similar to the reports of Siołek et al. and Kaczmarek-Ryś et al. [15,17]. 

This work also has some limitations. Our study included mainly low risk (62.1%) tumors, with a large number of microcarcinomas (≤1 cm, 57.4%) and it may have an impact on our results, similarly to other studies [7,35,42,56]. However, the large number of microcarcinomas in new PTC cases is observed worldwide, mainly due to overdiagnosis [1,2,3,57]. Another limitation of the study is lack of *RAS* mutations assessment. Due to funding restrictions (this research received no specific grant from any funding agency) we were not able to test *RAS* genes for mutations. *RAS* mutations are detected in 10%–20% of PTC cases [58]. It is possible that the *CHEK2* protein counteracts the activation of the MAPK pathway induced by mutations in *RAS*. *RAS* mutated thyroid tumors more often have poor clinical course [59]. At present we can only speculate based on our data related to the group *BRAF*^V600E^/*CHEK2* WT patients (e.g. most of aggressive histologic variants in Table 3 are in this group; n = 7) that some cases may have *RAS*. Then it will be interesting to evaluate *RAS* mutation in our material in future studies. Our study also lacks the *TERT* promoter mutations assessment. However, the prognostic role of *TERT* promoter mutation in the PTC has already been described by Maggisano et al. who concluded that *TERT* promoter may represent an excellent therapeutic target in subgroups of aggressive PTCs [60]. In another study conducted by Moon et al., the coexistence of *TERT* promoter mutations and *BRAF*^V600E^ has been found to have synergistic effect on clinical outcome in PTC, whereas the mutations alone had a modest effect [61]. This was confirmed in another study by Vuong et al [62]. 

Another limitation of our study could be a relatively small number of cases in some groups. However, the data presented in our work are nevertheless interesting, because they come from one center and are characterized by long follow-up of patients and may be used in the future for possible meta-analyzes.

To date, there have been no reports in the literature assessing the coexistence of the *BRAF*^V600E^ somatic mutation and germline mutations in *CHEK2* in patients with PTC. These types of mutation coexisted in 10.8% of our samples, and more than half of patients who had a mutation in *CHEK2* also had tumors with the *BRAF*^V600E^ mutation. *BRAF*^V600E^ was associated with some clinicopathological features, such as older age, extrathyroidal extension, and classic PTC, while *CHEK2* mutations were associated with older age and classic PTC. Coexistence of *CHEK2* mutation and *BRAF*^V600E^ was not a risk factor for a more aggressive disease. Hence, while the defect in DNA repair caused by *CHEK2* alteration may be a mechanism for the development of a more aggressive course in PTC, according to TCGA 2014 [16], our data do not support such a dependence.

## 5. Conclusions

We found that the coexistence of *BRAF*^V600E^ and mutations in *CHEK2* is a relatively frequent event in PTC and that more than half of patients who have a mutation in *CHEK2* also carry the *BRAF*^V600E^ alteration. Coexistence of both mutations is not associated with more aggressive clinicopathological features of PTC, poorer treatment response, or disease outcomes.

## Figures and Tables

**Table 1:** Characteristics of 427 patients with papillary thyroid carcinoma (PTC) and their *BRAF*^V600E^ and *CHEK2* mutation status.

Feature	Total n = 427 (100%)
**Sex**	
Female	377 (88.3%)
Male	50 (11.7%)
Age at diagnosis (years) *	
<55	278 (65.1%)
≥55	149 (34.9%)
Mean (SD)	48.5 (12.3)
Median (Q1–Q3; range)	50 (40–57; 15–76)
**Tumor diameter (mm)**	
Mean (SD)	13.5 (12.6)
Median (Q_1_–Q_3_; range)	9 (6–17.7; 1.0–80)
**Tumor diameter (mm)**	
≤10	245 (57.4%)
>10–20	96 (22.5%)
>20	86 (20.1%)
**Papillary cancer histologic variant**	
Classic	353 (82.7%)
Follicular	61 (14.3%)
Other, non-aggressive	4 (0.9%)
Other, aggressive **	9 (2.1%)
**Multifocality**	100 (23.4%)
Nodal metastases *	
N0a	201 (47.1%)
N0b	178 (41.7%)
N1	48 (11.2%)
**Distant metastases (M1)**	4 (0.9%)
**Extrathyroidal extension**	
Negative	302 (70.7%)
Microscopic	125 (29.3%)
Gross	0 (0.0%)
**Vascular invasion**	
No	409 (95.8%)
Yes	18 (4.2%)
Tumor stage *	
T1	336 (78.7%)
T2	67 (15.7%)
T3	21 (4.9%)
T4	3 (0.7%)
TNM stage *	
I	403 (94.4%)
II	20 (4.7%)
III	2 (0.5%)
IV	2 (0.5%)
**Mutation status**	
*BRAF* ^V600E^	274 (64.2%)
*BRAF*^V600E^ only	228 (53.4%)
*CHEK2*	65 (15.2%)
*CHEK2* only	19 (4.4%)
*BRAF*^V600E^ + *CHEK2*	46 (10.8%)
*BRAF^V600E^*/*CHEK2* WT	134 (31.4%)
***CHEK2* truncating mutation**	
IVS2+1G > A	3 (4.6%)
Del5395	5 (7.7%)
1100delC	0 (0.0%)
***CHEK2* missense mutation**	
I157T (including 2 homozygotes)	56 (86.2%)
*CHEK2* truncating + missense mutations (I157T+ IVS2+1G > A)	1 (1.5%)
***CHEK2* mutation only**	19 (4.4%)
***CHEK2* truncating mutation**	
IVS2+1G > A	0 (0%)
Del5395	3 (15.8%)
1100delC	0 (0%)
***CHEK2* missense mutation**	
I157T	16 (84.2%)
***CHEK2* missense + truncating mutations (I157T+IVS2+1G > A)**	0 (0%)
**ATA initial risk stratification system**	
Low	265 (62.1%)
Intermediate	151 (35.4%)
High	11 (2.6%)
**Radioactive iodine** **(** **I-131** **) therapy**	
No	101 (23.7%)
Yes	326 (76.3%)
**Response to therapy**	
Excellent	363 (85%)
Indeterminate	46 (10.8%)
Biochemically incomplete	7 (1.6%)
Structurally incomplete	11 (2.6%)
**Follow-up, recurrence**	4 (0.9%)
**Final follow-up (31 October 2018)**	
NED	408 (95.6%)
Biochemically persistent disease	16 (3.7%)
Structurally persistent disease	3 (0.7%)
Death	0 (0%)
**Follow-up (years)**	
Median (range)	10 (3–18)

SD, standard deviation; NED, no evidence of disease; N0a, one or more cytologically or histologically confirmed benign lymph node; N0b, no radiologic or clinical evidence of locoregional lymph node metastasis; N1, metastasis to regional lymph nodes; ATA, American Thyroid Association. * Determined according to the 8th edition of the American Joint Committee on Cancer/Union for International Cancer Control TNM staging system. ** Oxyphilic, diffuse sclerosing, solid. *BRAF*^V600E^/*CHEK2* WT (wild type) – cases without the following mutations detected: *BRAF*^V600E^, I157T, 1100delC, IVS2+1G > A, del5395.

**Table 2:** Clinical characteristics of patients with PTC carrying two *CHEK2* mutations.

Feature	I157T Missense Mutation (Including 2 Homozygous Variants)		*CHEK2* Missense + Truncating Mutations (I157T+IVS2+1G > A)
**Sex**	Female	Male	Female
**Age at diagnosis**	57	66	60
**Tumor diameter (mm)**	10	32	19
**Papillary cancer histologic variant**	Classic	Classic	Classic
**Multifocality**	Yes	Yes	No
Nodal metastases *	N0a	N1	N0b
**Distant metastases**	No	No	No
**Extrathyroidal extension**	No	No	No
**Vascular invasion**	No	No	No
Tumor stage *	T1a	T2	T1b
TNM stage *	I	II	I
**Coexisting *BRAF*^V600E^ and *CHEK2* mutations**	Yes	Yes	Yes
**ATA initial risk stratification system**	Low	Intermediate	Low
**Radioactive iodine (** **I-131** **) therapy**	1 dose (2700 MBq)	1 dose (2700 MBq)	1 dose (2700 MBq)
**Response to therapy**	Excellent	Excellent	Excellent
**Follow-up: recurrence**	No	No	No
**Final follow-up (31 October 2018)**	NED	NED	NED
**Follow-up (years)**	9	7	7

NED, no evidence of disease; N0a, one or more cytologically or histologically confirmed benign lymph nodes; N0b, no radiologic or clinical evidence of locoregional lymph node metastasis; N1, metastasis to regional lymph nodes; ATA, American Thyroid Association; * Determined according to the 8th edition of the American Joint Committee on Cancer/Union for International Cancer Control TNM staging system.

**Table 3:** Impact of *BRAF*^V600E^ or *CHEK2* mutations or their coexistence on clinicopathological characteristics, response to therapy, and disease outcome.

Feature	*BRAF*^V600E^/*CHEK2* WT n = 134	*BRAF*^V600E^ Mutation Only n = 228	*CHEK2* Mutation Only n = 19	*BRAF*^V600E^*+ CHEK2* Mutation n = 46	*p*-Value ^1^			
					*BRAF*^V600E^/*CHEK2* WT vs. *BRAF*^V600E^	*BRAF*^V600E^/*CHEK2* WT vs. *CHEK2*	*BRAF*^V600E^/*CHEK2* WT vs. *BRAF*^V600E^ + *CHEK2*	*BRAF*^V600E^ + *CHEK2* vs. *BRAF*^V600E^
**Sex**					0.669	0.431	0.952	0.729
Female	119 (88.8%)	199 (87.3%)	18 (94.7%)	41 (89.1%)				
Male	15 (11.2%)	29 (12.7%)	1 (5.3%)	5 (10.9%)				
Age at diagnosis (years) *					0.099	0.094	0.175	0.770
<55	96 (71.6%)	144 (63.2%)	10 (52.6%)	28 (60.9%)				
≥55	38 (28.4%)	84 (36.8%)	9 (47.4%)	18 (39.1%)				
Mean (SD)	45.8 (13.1)	49.5 (11.9)	52.8 (10.5)	49.7 (11.5)				
Median (Q_1_–Q_3_)	47 (36–56)	51 (41–58)	54 (46–60)	50 (42–58)				
Range	25–76	19–74	32–70	18–71	0.016	0.028	0.072	0.775
**Tumor diameter (mm)**					0.517	0.247	0.465	0.215
Mean (SD)	13.9 (12.7)	13.1 (12.3)	11.2 (11.3)	15.4 (14.4)				
Median (Q_1_–Q_3_)	10 (6–20)	9 (6–16)	7 (4.2–14.5)	10 (7–21)				
Range	1.0–80	1.5–75	2.0–50	1.0–80				
**Tumor diameter (mm)**					0.608	0.549	0.763	0.524
≤10	74 (55.2%)	138 (60.5%)	13 (68.4%)	24 (52.2%)				
>10–20	32 (23.9%)	47 (20.6%)	3 (15.8%)	10 (21.7%)				
>20	28 (20.9%)	43 (18.9%)	3 (15.8%)	12 (26.1%)				
**Papillary cancer histologic variant**					0.003	0.027	0.101	0.715
Classic	99 (73.9%)	197 (86.4%)	16 (84.2%)	41 (89.1%)				
Follicular	28 (20.9%)	27 (11.8%)	2 (10.5%)	4 (8.7%)				
Other, non-aggressive	0 (0.0%)	2 (0.9%)	1 (5.3%)	1 (2.2%)				
Other, aggressive **	7 (5.2%)	2 (0.9%)	0 (0.0%)	0 (0.0%)				
**Multifocality**	24 (17.9%)	61 (26.8%)	5 (26.3%)	10 (21.7%)	0.056	0.383	0.568	0.479
Nodal metastases *					0.495	0.736	0.782	0.443
N0a	62 (46.3%)	109 (47.8%)	7 (36.8%)	23 (50.0%)				
N0b	59 (44.0%)	89 (39.0%)	10 (52.6%)	20 (43.5%)				
N1	13 (9.7%)	30 (13.2%)	2 (10.5%)	3 (6.5%)				
**Distant metastases (M1)**	4 (3.0%)	0 (0.0%)	0 (0.0%)	0 (0.0%)	0.009	0.447	0.237	-
**Extrathyroidal extension**					0.039	0.191	0.556	0.435
Negative	102 (76.1%)	150 (65.8%)	17 (89.5%)	33 (71.7%)				
Microscopic	32 (23.9%)	78 (34.2%)	2 (10.5%)	13 (28.3%)				
**Vascular invasion**					0.429	0.994	0.815	0.782
No	127 (94.8%)	220 (96.5%)	18 (94.7%)	44 (95.7%)				
Yes	7 (5.2%)	8 (3.5%)	1 (5.3%)	2 (4.3%)				
Tumor stage *					0.655	0.893	0.601	0.724
T1	104 (77.6%)	182 (79.8%)	16 (84.2%)	34 (73.9%)				
T2	20 (14.9%)	35 (15.4%)	2 (10.5%)	10 (21.7%)				
T3	8 (6.0%)	10 (4.4%)	1 (5.3%)	2 (4.3%)				
T4	2 (1.5%)	1 (0.4%)	0 (0.0%)	0 (0.0%)				
TNM stage *					0.286	0.543	0.784	0.894
I	126 (94.0%)	216 (94.7%)	17 (89.5%)	44 (95.7%)				
II	5 (3.7%)	11 (4.8%)	2 (10.5%)	2 (4.3%)				
III	1 (0.7%)	1 (0.4%)	0 (0.0%)	0 (0.0%)				
IV	2 (1.5%)	0 (0.0%)	0 (0.0%)	0 (0.0%)				
**ATA initial risk stratification system**					0.065	0.119	0.904	0.176
Low	89 (66.4%)	129 (56.6%)	16 (84.2%)	31 (67.4%)				
Intermediate + high	45 (33.6%)	99 (43.4%)	3 (15.8%)	15 (32.6%)				
**Radioactive iodine (** **I-131** **) therapy**					0.927	0.169	0.609	0.632
No	30 (22.4%)	52 (22.8%)	7 (36.8%)	12 (26.1%)				
Yes	104 (77.6%)	176 (77.2%)	12 (63.2%)	34 (73.9%)				
**Response to therapy**					0.304	0.090	0.947	0.457
Excellent	116 (86.6%)	188 (82.5%)	19 (100.0%)	40 (87.0%)				
Other than excellent ***	18 (13.4%)	40 (17.5%)	0 (0.0%)	6 (13.0%)				
**Follow-up: recurrence**	0 (0.0%)	4 (1.8%)	0 (0.0%)	0 (0.0%)	0.124	N/A	N/A	0.366
**Status at final follow-up**					0.401	0.642	0.675	0.652
Remission (NED)	128 (95.5%)	216 (94.7%)	19 (100.0%)	45 (97.8%)				
Biochemically persistent disease	4 (3.0%)	11 (4.8%)	0 (0.0%)	1 (2.2%)				
Structurally persistent disease	2 (1.5%)	1 (0.4%)	0 (0.0 %)	0 (0.0 %)				
**Follow-up (years)** **Median range**	11 (3–18)	9 (3–17)	11 (5–18)	9 (3–18)	0.012	0.974	0.239	0.894

SD, standard deviation; NED, no evidence of disease; N0a, one or more cytologically or histologically confirmed benign lymph node; N0b, no radiologic or clinical evidence of locoregional lymph node metastasis; N1, metastasis to regional lymph nodes; ATA, American Thyroid Association; ^1^ For categorical variable the Bonferroni correction was performed; the Bonferroni correction compensates for that increase by testing each individual hypothesis at a significance level of a/m, where "a" is the desired overall alpha level and "m" is the number of hypotheses. In this table, the trial is testing m = 4 hypotheses with a desired a = 0.05, so the Bonferroni correction for *p*-value < 0.0125 [0.05/4] is considered as significant; * Determined according to the 8th edition of the American Joint Committee on Cancer/Union for International Cancer Control TNM staging system; ** Oxyphilic, diffuse sclerosing, solid; *** Indeterminate, biochemically incomplete, structurally incomplete; *BRAF*^V600E^/*CHEK2* WT (wild type)—cases without the following mutations detected: *BRAF^V600E^*, I157T, 1100delC, IVS2+1G > A, del5395.
